# Association of Neurocognitive and Physical Function With Gait Speed in Midlife

**DOI:** 10.1001/jamanetworkopen.2019.13123

**Published:** 2019-10-11

**Authors:** Line Jee Hartmann Rasmussen, Avshalom Caspi, Antony Ambler, Jonathan M. Broadbent, Harvey J. Cohen, Tracy d’Arbeloff, Maxwell Elliott, Robert J. Hancox, HonaLee Harrington, Sean Hogan, Renate Houts, David Ireland, Annchen R. Knodt, Kim Meredith-Jones, Miriam C. Morey, Lynda Morrison, Richie Poulton, Sandhya Ramrakha, Leah Richmond-Rakerd, Maria L. Sison, Kate Sneddon, W. Murray Thomson, Ahmad R. Hariri, Terrie E. Moffitt

**Affiliations:** 1 Department of Psychology and Neuroscience, Duke University, Durham, North Carolina; 2 Clinical Research Centre, Copenhagen University Hospital Amager and Hvidovre, Hvidovre, Denmark; 3 Department of Psychiatry and Behavioral Sciences, Duke University School of Medicine, Durham, North Carolina; 4 Center for Genomic and Computational Biology, Duke University, Durham, North Carolina; 5 Social, Genetic, and Developmental Psychiatry Centre, Institute of Psychiatry, Psychology, and Neuroscience, King’s College London, London, United Kingdom; 6 Dunedin Multidisciplinary Health and Development Research Unit, Department of Psychology, University of Otago, Dunedin, New Zealand; 7 Department of Oral Sciences, University of Otago, Dunedin, New Zealand; 8 Claude D. Pepper Older Americans Independence Center, Duke University, Durham, North Carolina; 9 Duke Center for the Study of Aging and Human Development, Duke University, Durham, North Carolina; 10 Department of Medicine, Duke University, Durham, North Carolina; 11 Department of Preventive and Social Medicine, Dunedin School of Medicine, University of Otago, Dunedin, New Zealand; 12 Geriatric Research, Education, and Clinical Center, Durham VA Medical Center, Durham, North Carolina; 13 Frank Porter Graham Child Development Institute, University of North Carolina at Chapel Hill, Chapel Hill

## Abstract

**Importance:**

Gait speed is a well-known indicator of risk of functional decline and mortality in older adults, but little is known about the factors associated with gait speed earlier in life.

**Objectives:**

To test the hypothesis that slow gait speed reflects accelerated biological aging at midlife, as well as poor neurocognitive functioning in childhood and cognitive decline from childhood to midlife.

**Design, Setting, and Participants:**

This cohort study uses data from the Dunedin Multidisciplinary Health and Development Study, a population-based study of a representative 1972 to 1973 birth cohort in New Zealand that observed participants to age 45 years (until April 2019). Data analysis was performed from April to June 2019.

**Exposures:**

Childhood neurocognitive functions and accelerated aging, brain structure, and concurrent physical and cognitive functions in adulthood.

**Main Outcomes and Measures:**

Gait speed at age 45 years, measured under 3 walking conditions: usual, dual task, and maximum gait speeds.

**Results:**

Of the 1037 original participants (91% of eligible births; 535 [51.6%] male), 997 were alive at age 45 years, of whom 904 (90.7%) had gait speed measured (455 [50.3%] male; 93% white). The mean (SD) gait speeds were 1.30 (0.17) m/s for usual gait, 1.16 (0.23) m/s for dual task gait, and 1.99 (0.29) m/s for maximum gait. Adults with more physical limitations (standardized regression coefficient [β], −0.27; 95% CI, −0.34 to −0.21; *P* < .001), poorer physical functions (ie, weak grip strength [β, 0.36; 95% CI, 0.25 to 0.46], poor balance [β, 0.28; 95% CI, 0.21 to 0.34], poor visual-motor coordination [β, 0.24; 95% CI, 0.17 to 0.30], and poor performance on the chair-stand [β, 0.34; 95% CI, 0.27 to 0.40] or 2-minute step tests [β, 0.33; 95% CI, 0.27 to 0.39]; all *P* < .001), accelerated biological aging across multiple organ systems (β, −0.33; 95% CI, −0.40 to −0.27; *P* < .001), older facial appearance (β, −0.25; 95% CI, −0.31 to −0.18; *P* < .001), smaller brain volume (β, 0.15; 95% CI, 0.06 to 0.23; *P* < .001), more cortical thinning (β, 0.09; 95% CI, 0.02 to 0.16; *P* = .01), smaller cortical surface area (β, 0.13; 95% CI, 0.04 to 0.21; *P* = .003), and more white matter hyperintensities (β, −0.09; 95% CI, −0.15 to −0.02; *P* = .01) had slower gait speed. Participants with lower IQ in midlife (β, 0.38; 95% CI, 0.32 to 0.44; *P* < .001) and participants who exhibited cognitive decline from childhood to adulthood (β, 0.10; 95% CI, 0.04 to 0.17; *P* < .001) had slower gait at age 45 years. Those with poor neurocognitive functioning as early as age 3 years had slower gait in midlife (β, 0.26; 95% CI, 0.20 to 0.32; *P* < .001).

**Conclusions and Relevance:**

Adults’ gait speed is associated with more than geriatric functional status; it is also associated with midlife aging and lifelong brain health.

## Key Points

**Question:**

Is gait speed measured at age 45 years associated with accelerated biological aging, neurocognitive function, and cognitive decline?

**Findings:**

In this 5-decade cohort study of 904 participants in New Zealand, physical and biological indicators of accelerated aging, including compromised brain integrity (eg, reduced brain volume and cortical thickness), were associated with slow gait measured at age 45 years. Lifelong compromised brain health—including poor neurocognitive functioning as early as age 3 years and childhood-to-adulthood decline in cognitive functioning—was associated with slower gait at midlife.

**Meaning:**

Gait speed at midlife may be a summary index of lifelong aging with possible origins in childhood central nervous system deficits.

## Teaser

This 5-decade cohort study of a New Zealand population examines whether slow gait speed in middle age is associated with accelerated biological aging, as well as poor neurocognitive functioning in childhood and cognitive decline from childhood to middle age.

## Introduction

The ability to walk and gait speed depend on the function and interplay of the musculoskeletal, visual, central nervous, and peripheral nervous systems, as well as aerobic capacity, cardiorespiratory fitness, and energy production and delivery.^1,2^ Reduced gait speed is a sign of advancing age^3^; it is associated with poorer response to rehabilitation, age-related diseases, including cardiovascular disease and dementia, and early mortality.^4,5,6^

Gait speed is frequently used in geriatric settings as a quick, simple, and reliable way of estimating older patients’ functional capacity. It is increasingly recognized that gait is associated with not only musculoskeletal mechanisms but also with the central nervous system (CNS).^4,7^ To date, longitudinal research on gait and cognitive functioning has primarily focused on older adults, many with neurological diseases.^8^ Few studies have integrated cognitive and structural measures of the CNS with gait in healthy midlife adults, and, to our knowledge, none has examined the childhood CNS origins of gait. Filling this information gap is important for understanding the origins of gait speed and for prevention of functional disability. If gait speed is antedated by early-life CNS variation, this would point to possibilities for early identification of vulnerability and resilience in functional capacity well before late life and suggest potential targets for early intervention.

Herein, we evaluated 2 hypotheses. First, we tested the hypothesis that slow gait speed at midlife—when adults are still in their robust 40s—already reflects early signs of accelerated biological aging. If so, this would imply that gait speed could be used as an earlier indicator of aging in aging-prevention trials. Second, we tested the hypothesis that slow gait speed is associated with poor neurocognitive functioning at midlife and also in early childhood. If so, this would imply that gait speed has origins in brain development beginning in childhood and manifesting in midlife. Support for our hypotheses would suggest rethinking gait speed, from a geriatric index of adult functional decline to a summary index of lifelong aging with possible origins in childhood CNS deficits.

## Methods

### Study Design and Population

Participants are members of the Dunedin Multidisciplinary Health and Development Study, a longitudinal investigation of health and behavior in a representative birth cohort. The 1037 participants (91% of eligible births) were all individuals born between April 1972 and March 1973 in Dunedin, New Zealand, who were eligible on the basis of residence in the province and who participated in the first assessment at age 3 years.^9^ The cohort represents the full range of socioeconomic status (SES) in the general population of New Zealand’s South Island and, as adults, matches the New Zealand National Health and Nutrition Survey on key adult health indicators (eg, body mass index, smoking, and general practitioner visits) and the New Zealand Census of citizens of the same age on educational attainment. Participants are primarily white (93%, self-identified), matching South Island demographic characteristics.^9^ Assessments were performed at birth; at ages 3, 5, 7, 9, 11, 13, 15, 18, 21, 26, 32, and 38 years; and, most recently (completed April 2019), at age 45 years, when 938 of the 997 participants (94.1%) still alive participated. At each assessment, each participant was brought to the research unit for interviews and examinations. Written informed consent was obtained from cohort participants, and study protocols were approved by the institutional ethical review boards of the participating universities. This study follows the Strengthening the Reporting of Observational Studies in Epidemiology (STROBE) reporting guideline.

### Gait Speed

Gait speed (meters per second) was assessed with the 6-m-long GAITRite Electronic Walkway (CIR Systems, Inc) with 2-m acceleration and 2-m deceleration before and after the walkway, respectively. We excluded 4 participants who could not be tested because of disabling conditions (eg, amputation or broken leg). Gait speed was assessed under 3 walking conditions: usual gait speed (walk at normal pace from a standing start, measured as a mean of 2 walks) and 2 challenge paradigms, dual task gait speed (walk at normal pace while reciting alternate letters of the alphabet out loud, starting with the letter “A,” measured as a mean of 2 walks) and maximum gait speed (walk as fast as safely possible, measured as a mean of 3 walks).

### Composite Gait Speed

**Figure 1.**
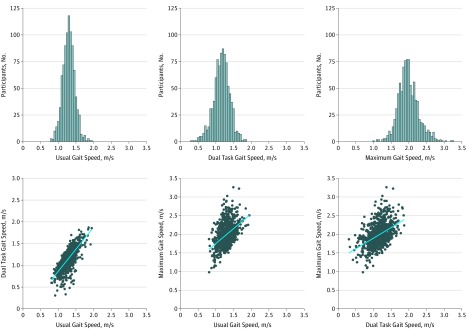
Distribution of Gait Speed for Participants in the Dunedin Multidisciplinary Health and Development Study at Age 45 Years Gait speed distributions for individual walk conditions (usual, dual task, and maximum) are depicted as histograms. Scatterplots illustrate the pairwise correlations between individual walk conditions. The blue lines are linear regression lines.

Gait speed was correlated across the 3 walk conditions: usual vs dual task, usual vs maximum, and dual task vs maximum (Figure 1). To increase reliable measurements and take advantage of the variation in all 3 walk conditions (usual gait and the 2 challenge paradigms), we calculated the mean of the 3 individual walk conditions to generate our primary measure of composite gait speed (eFigure 1 in the Supplement).

### Physical Function

Physical function at age 45 years was assessed by self-reported physical limitations using the RAND 36-Item Short Form Survey,^10^ with reversed scores to reflect limitations. Physical function was also assessed by several brief exercises that index the ability to perform everyday activities, including handgrip strength,^11,12^ balance,^13,14,15^ visual-motor coordination,^16^ chair-stand test,^17,18^ and 2-minute step test^18,19^ (eMethods 1 in the Supplement).

### Measures of Accelerated Aging

Accelerated aging was assessed by 2 measures: pace of aging^20^ and facial age^20^ (eMethods 1 in the Supplement). The pace of aging was measured for each participant with repeated assessments of a panel of 19 biomarkers taken at ages 26, 32, 38, and 45 years.^20^ The 19 biomarkers were body mass index, waist-to-hip ratio, glycated hemoglobin level, leptin level, blood pressure (mean arterial pressure), cardiorespiratory fitness (maximum oxygen consumption [VO_2_max]), forced expiratory volume in 1 second (FEV_1_), ratio of forced expiratory volume in 1 second to forced vital capacity (FEV_1_/FVC), total cholesterol level, triglyceride level, high-density lipoprotein cholesterol level, ratio of apolipoprotein B100 to apolipoprotein A1, lipoprotein(a) level, creatinine clearance, blood urea nitrogen level, C-reactive protein level, white blood cell count, gum health, and caries-affected tooth surfaces. Change over time in each biomarker was modeled with mixed-effects growth models, and these rates of change were combined into a single index scaled (by sex) in years of physiological change occurring per 1 chronological year. Participants ranged in their pace of aging from approximately 0 years of physiological change per chronological year to approximately 3 years of physiological change per chronological year. Facial age was evaluated on the basis of ratings by an independent panel of 8 raters of standardized photographs of each participant’s face made during their assessment at age 45 years.

### Brain Structure and Neurocognitive Functions

At age 45 years, participants completed a neuroimaging protocol to detect structural age-related features of the brain. Images (T1-weighted structural and fluid-attenuated inversion recovery) were acquired using a 3-T magnetic resonance imaging scanner (Skyra; Siemens Healthcare) equipped with a 64-channel head and neck coil. High-resolution structural images were used to generate estimates of total brain volume, mean cortical thickness, total surface area, and white matter hyperintensities (eMethods 1 in the Supplement). Total white matter hyperintensities were log-transformed (natural logarithm) to improve normality.

Neurocognitive function at age 45 years was assessed with the Wechsler Adult Intelligence Scale–IV.^21^ The Wechsler Adult Intelligence Scale–IV generates the overall full-scale IQ. In addition, 4 Wechsler Adult Intelligence Scale–IV indexes assess the abilities that make up the IQ: processing speed, working memory, perceptual reasoning, and verbal comprehension. In addition, the Trail-Making Test,^22^ Animal Naming Test, Wechsler Memory Scale–Mental Control, and the Rey Auditory Verbal Learning Test of Memory^16^ were administered at age 45 years to assess executive functioning, verbal fluency, and memory (eMethods 1 in the Supplement). Trail-Making Test scores were reversed so that higher values corresponded to better cognitive performance.

### Measures of Childhood Neurocognitive Functions and Childhood SES

At age 3 years, each child participated in a 45-minute examination that included assessments by a pediatric neurologist; standardized tests of intelligence, receptive language, and motor skills; and examiner ratings of each child’s emotional and behavioral regulation. These 5 measures were combined to yield a composite index of brain health (eMethods 2 in the Supplement).^23^ Childhood-to-adulthood cognitive decline was calculated by a difference between scores on the Wechsler Adult Intelligence Scales and the Wechsler Intelligence Scale for Children–Revised.^24^ These tests are ideal for measuring childhood-to-adulthood cognitive decline because both tests are matched for content coverage and format, both were individually administered by trained psychometrists, and both yield summary scores that are reliable at greater than 0.95. Mean scores for the Wechsler Intelligence Scale for Children–Revised across administration at ages 7, 9, and 11 years were calculated (eMethods 2 in the Supplement). The SES of participants’ childhood families was measured using the 6-point Elley-Irving Socioeconomic Index for New Zealand.^25^

### Statistical Analysis

Continuous measures are presented as mean (SD) or median (interquartile range). We calculated Pearson correlation coefficients (*r*). We performed linear regression analyses with all variables standardized to mean = 0 and SD = 1, and we present standardized regression coefficients (β) for the associations between individual factors with gait speed, adjusted for sex. Associations were further adjusted for leg length, body composition (fat mass and lean mass), or childhood SES. These results are presented in eTables 1, 2, 3, and 4 in the Supplement; further details about the measurement of leg length, fat mass index, and lean mass index are provided in eMethods 1 in the Supplement. We applied Bonferroni correction to account for multiple testing within domain sets of measures (physical function, accelerated aging, brain structure, and neurocognitive function).

Statistical analyses were performed in SAS Enterprise Guide statistical software version 7.15 (SAS Institute). Analyses reported here were checked for reproducibility by an independent data analyst, who recreated the code by working from the manuscript and applied it to a fresh copy of the data set. Two-sided *P* < .05 (Fisher exact test) was a priori designated as statistically significant. We present effect sizes, 95% CIs, and actual *P* values for all tests conducted. Data analysis was performed from April to June 2019.

## Results

Of 1037 participants in the original cohort (535 [51.6%] male), 997 were still alive at age 45 years, and 938 took part in the assessment at age 45 years between April 2017 and April 2019. Of the 997 still alive, 904 (90.7%; 455 [50.3%] male; 93% white) completed the gait test and were included in this study. Participants with gait speed data available did not differ significantly from other living participants in terms of childhood SES or childhood neurocognitive functioning (see attrition analysis in eMethods 3 in the Supplement). Table 1 shows the demographic characteristics and mean (SD) for measures of gait speed, physical function (mean [SD], physical limitation score, 10.2 [15.4]; maximum handgrip strength, 39.8 [12.0] kg; 1-legged balance, 14.8 [9.8] seconds; visual-motor coordination, 71.4 [12.6] seconds; number of chair stands in 30 seconds, 18.3 [5.6]; and 2-minute step test, 115.5 [26.6] steps), accelerated aging (mean [SD], pace of aging score, 0.99 [0.31]; facial age score, −0.004 [1.00]), brain structure (mean [SD], total brain volume, 1 160 304.5 [116 687.8] mm^3^; mean cortical thickness, 2.56 [0.09] mm; total surface area, 185 514.9 [16 350.8] mm^2^; and total log-transformed white matter hyperintensities, 936.2 [1050.8] mm^3^), and neurocognitive function (mean [SD], childhood brain health *z* score, 0.05 [0.93]; total IQ, 100.1 [14.9]). Gait speed was normally distributed under all walk conditions, with larger variation in gait speed during the dual task and maximum walk conditions (usual vs dual task, *r* = 0.75 [95% CI, 0.72-0.77], *P* < .001; usual vs maximum, *r* = 0.46 [95% CI, 0.41-0.51], *P* < .001; and dual task vs maximum, *r* = 0.45 [95% CI, 0.40-0.50], *P* < .001) (Figure 1). The mean (SD) usual gait speed was 1.30 (0.17) m/s; dual task gait speed, 1.16 (0.23) m/s; and maximum gait speed, 1.99 (0.29) m/s. The mean (SD) composite gait speed was 1.48 (0.19) m/s. The median (interquartile range) usual gait speed was 1.30 (1.18-1.40) m/s; dual task gait speed, 1.17 (1.03-1.31) m/s; maximum gait speed, 1.96 (1.80-2.15) m/s; and composite gait speed, 1.48 (1.35-1.60) m/s. One-week gait speed test-retest reliabilities (50 participants) were *r* = 0.77 (95% CI, 0.62-0.86; *P* < .001) for usual gait speed, *r* = 0.86 (95% CI, 0.75-0.91; *P* < .001) for dual task gait speed, *r* = 0.74 (95% CI, 0.58-0.84; *P* < .001) for maximum gait speed, and *r* = 0.77 (95% CI, 0.62-0.86; *P* < .001) for composite gait speed. Later in the article, we describe associations between composite gait speed and measures in domains of physical function, accelerated aging, brain structure, and neurocognitive function. Each of the 3 individual walk conditions yielded the same pattern of associations as did composite gait speed (Table 2; eTable 3 and eTable 4 in the Supplement). All associations were independent of leg length and body composition (lean mass and fat mass) (eTable 1 and eTable 2 in the Supplement) as well as childhood SES (eTable 3 and eTable 4 in the Supplement), except white matter hyperintensities, which became nonsignificant when we controlled for body composition and childhood SES. When correcting for multiple testing within each domain, all measures remained significantly associated with gait speed.

**Table 1.** Characteristics of Participants With Gait Speed Data

Characteristic	Participants, No. (N = 904)^a^	Mean (SD)
Childhood socioeconomic status score	899	3.78 (1.13)
Gait speed, m/s		
Usual	904	1.30 (0.17)
Dual task	904	1.16 (0.23)
Maximum	904	1.99 (0.29)
Composite	904	1.48 (0.19)
Physical function		
Physical limitation score^b^	901	10.2 (15.4)
Maximum handgrip strength, kg	903	39.8 (12.0)
One-legged balance, s	897	14.8 (9.8)
Visual-motor coordination, s^c^	899	71.4 (12.6)
Chair stands, No. in 30 s	873	18.3 (5.6)
2-min step test, No. of steps	886	115.5 (26.6)
Accelerated aging		
Pace of aging score^d^	903	0.99 (0.31)
Facial age score	902	−0.004 (1.00)
Brain structure		
Total brain volume, mm^3^	859	1 160 304.5 (116 687.8)
Mean cortical thickness, mm	859	2.56 (0.09)
Total surface area, mm^2^	859	185 514.9 (16 350.8)
Total log-transformed white matter hyperintensities, mm^3^^e^	849	936.2 (1050.8)
Neurocognitive function		
Childhood brain health *z *score	902	0.05 (0.93)
Total IQ	902	100.1 (14.9)
Processing speed	902	100.1 (15.0)
Working memory	898	100.1 (15.0)
Perceptual reasoning	902	100.1 (14.9)
Verbal comprehension	892	100.0 (15.0)
Trail-Making Test, s		
Part A	901	30.2 (9.9)
Part B	902	68.4 (22.5)
Animal Naming Test, No. in 60 s	895	23.4 (5.8)
Wechsler Memory Scale–Mental Control score^f^	888	3.05 (1.34)
Rey Auditory Verbal Learning test score		
Total	902	35.7 (7.4)
Recall	898	8.7 (2.9)

^a^ A total of 455 participants (50.3%) were male.

^b^ Measured according to the RAND 36-Item Short Form Survey physical functioning scale with reversed scores to reflect limitations.^10^

^c^ Grooved pegboard test, time (seconds) for nondominant hand.

^d^ Years of physiological change per chronological year.

^e^ Natural logarithm.

^f^ Naming the months backward.

**Table 2.** Associations of Gait Speed With Concurrent Measures of Physical Function, Accelerated Aging, Brain Structure, and Neurocognitive Function^a^

Variable	Participants, No.	Gait Speed							
		Usual		Dual Task		Maximum		Composite	
		β (95% CI)	*P* Value	β (95% CI)	*P* Value	β (95% CI)	*P* Value	β (95% CI)	*P* Value
Physical function									
Physical limitations^b^	901	−0.21 (−0.27 to −0.15)	<.001	−0.19 (−0.25 to −0.12)	<.001	−0.27 (−0.34 to −0.21)	<.001	−0.27 (−0.34 to −0.21)	<.001
Maximum handgrip strength, kg	903	0.17 (0.06 to 0.28)	.002	0.24 (0.13 to 0.34)	<.001	0.41 (0.31 to 0.52)	<.001	0.36 (0.25 to 0.46)	<.001
One-legged balance, s	897	0.17 (0.10 to 0.23)	<.001	0.19 (0.12 to 0.25)	<.001	0.30 (0.24 to 0.36)	<.001	0.28 (0.21 to 0.34)	<.001
Visual-motor coordination^c^	899	0.12 (0.06 to 0.19)	<.001	0.20 (0.14 to 0.27)	<.001	0.24 (0.17 to 0.30)	<.001	0.24 (0.17 to 0.30)	<.001
Chair stands, No. in 30 s	873	0.21 (0.15 to 0.28)	<.001	0.23 (0.17 to 0.30)	<.001	0.36 (0.30 to 0.42)	<.001	0.34 (0.27 to 0.40)	<.001
2-min step test, No.	886	0.18 (0.11 to 0.24)	<.001	0.21 (0.15 to 0.28)	<.001	0.39 (0.32 to 0.44)	<.001	0.33 (0.27 to 0.39)	<.001
Accelerated aging									
Pace of aging^d^	903	−0.27 (−0.33 to −0.20)	<.001	−0.26 (−0.32 to −0.20)	<.001	−0.30 (−0.36 to −0.24)	<.001	−0.33 (−0.40 to −0.27)	<.001
Facial age	902	−0.18 (−0.25 to −0.12)	<.001	−0.17 (−0.23 to −0.10)	<.001	−0.25 (−0.31 to −0.19)	<.001	−0.25 (−0.31 to −0.18)	<.001
Brain structure									
Total brain volume, mm^3^	859	0.10 (0.01 to 0.18)	.02	0.13 (0.04 to 0.21)	.004	0.14 (0.05 to 0.22)	.002	0.15 (0.06 to 0.23)	<.001
Mean cortical thickness, mm	859	0.06 (−0.003 to 0.13)	.06	0.07 (0.01 to 0.14)	.03	0.08 (0.01 to 0.15)	.02	0.09 (0.02 to 0.16)	.01
Total surface area, mm^2^	859	0.10 (0.01 to 0.18)	.02	0.11 (0.02 to 0.19)	.01	0.11 (0.02 to 0.19)	.01	0.13 (0.04 to 0.21)	.003
Total log-transformed white matter hyperintensities, mm^3^	849	−0.05 (−0.12 to 0.02)	.14	−0.08 (−0.15 to −0.01)	.02	−0.07 (−0.14 to −0.01)	.03	−0.09 (−0.15 to −0.02)	.01
Cognitive function									
Total IQ	902	0.23 (0.17 to 0.29)	<.001	0.29 (0.22 to 0.35)	<.001	0.39 (0.33 to 0.45)	<.001	0.38 (0.32 to 0.44)	<.001
Processing speed	902	0.19 (0.12 to 0.25)	<.001	0.27 (0.20 to 0.33)	<.001	0.26 (0.20 to 0.33)	<.001	0.30 (0.23 to 0.36)	<.001
Working memory	898	0.20 (0.14 to 0.27)	<.001	0.25 (0.19 to 0.32)	<.001	0.31 (0.25 to 0.38)	<.001	0.32 (0.26 to 0.38)	<.001
Perceptual reasoning	902	0.15 (0.08 to 0.21)	<.001	0.20 (0.14 to 0.27)	<.001	0.32 (0.26 to 0.38)	<.001	0.29 (0.22 to 0.35)	<.001
Verbal comprehension	892	0.19 (0.13 to 0.26)	<.001	0.19 (0.12 to 0.25)	<.001	0.34 (0.28 to 0.40)	<.001	0.30 (0.24 to 0.37)	<.001
Trail-Making Test^e^									
Part A	901	0.16 (0.10 to 0.23)	<.001	0.23 (0.17 to 0.29)	<.001	0.24 (0.18 to 0.31)	<.001	0.26 (0.20 to 0.33)	<.001
Part B	902	0.12 (0.05 to 0.18)	<.001	0.18 (0.11 to 0.24)	<.001	0.23 (0.17 to 0.30)	<.001	0.22 (0.16 to 0.29)	<.001
Animal Naming Test, No. in 60 s	895	0.14 (0.08 to 0.21)	<.001	0.16 (0.09 to 0.22)	<.001	0.24 (0.18 to 0.31)	<.001	0.23 (0.16 to 0.29)	<.001
Wechsler Memory Scale–Mental Control^f^	888	0.15 (0.08 to 0.22)	<.001	0.14 (0.07 to 0.20)	<.001	0.19 (0.13 to 0.26)	<.001	0.20 (0.13 to 0.26)	<.001
Rey Auditory Verbal Learning Test of Memory									
Total	902	0.21 (0.15 to 0.28)	<.001	0.24 (0.17 to 0.31)	<.001	0.34 (0.27 to 0.40)	<.001	0.33 (0.27 to 0.39)	<.001
Recall	898	0.15 (0.08 to 0.21)	<.001	0.15 (0.08 to 0.22)	<.001	0.23 (0.16 to 0.30)	<.001	0.22 (0.15 to 0.29)	<.001

^a^ Standardized regression coefficients (β) were adjusted for sex.

^b^ Measured according to the RAND 36-Item Short Form Survey physical functioning scale with reversed scores to reflect limitations.^10^

^c^ Grooved pegboard test, time (seconds) for nondominant hand. For the linear regression analyses, scores were reversed so that higher values corresponded to better performance.

^d^ Years of physiological change per chronological year.

^e^ Scores for the Trail-Making Tests were reversed so that higher values corresponded to better cognitive performance.

^f^ Naming the months backward.

### Gait Speed and Physical Function and Accelerated Aging at Age 45 Years

Midlife adults who self-reported more physical limitations in their day-to-day life had slower gait speed (β, −0.27; 95% CI, −0.34 to −0.21; *P* < .001). In addition, adults with weaker grip strength (β, 0.36; 95% CI, 0.25-0.46; *P* < .001), poorer balance (β, 0.28; 95% CI, 0.21-0.34; *P* < .001), and poorer visual-motor coordination (β, 0.24; 95% CI, 0.17-0.30; *P* < .001) and those who performed worse on the chair-stand (β, 0.34; 95% CI, 0.27-0.40; *P* < .001) or 2-minute step tests (β, 0.33; 95% CI, 0.27-0.39; *P* < .001) had slower gait (Table 2).

**Figure 2.**
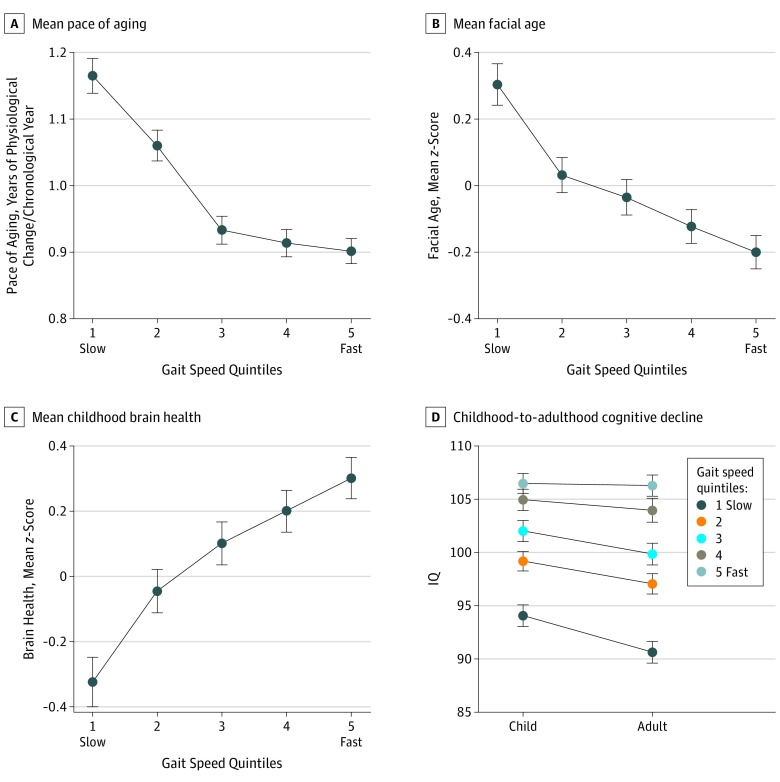
Accelerated Aging, Poor Childhood Neurocognitive Function, and Cognitive Decline Associated With Slower Midlife Gait Speed A-C, The mean pace of aging at age 45 years (years of physiological change per chronological year) (A), mean facial age at age 45 years (*z* score; mean = 0, SD = 1) (B), and mean brain health at age 3 years (*z* score; mean = 0, SD = 1) (C) by gait speed quintiles at age 45 years are shown. Generalized additive models are shown in eFigure 2 in the Supplement. D, The childhood-to-adulthood cognitive decline by gait speed quintiles is also shown. Gait speed quintiles are defined as follows: quintile 1, less than 1.32 m/s (181 participants); quintile 2, 1.32 to 1.43 m/s (181 participants); quintile 3, 1.44 to 1.52 m/s (181 participants); quintile 4, 1.53 to 1.63 m/s (181 participants); and quintile 5, greater than 1.63 m/s (180 participants). Error bars indicate standard error.

Midlife adults who exhibited signs of accelerated aging also had slower gait (Table 2). Slower gait was associated with a more rapid pace of aging (β, −0.33; 95% CI, −0.40 to −0.27; *P* < .001); according to the pace of aging index, participants with the slowest gait (bottom quintile; mean [SD] composite gait speed, 1.21 [0.10] m/s) had been aging 5.0 years faster from ages 26 to 45 years than participants with the fastest gait (top quintile; mean [SD] composite gait speed, 1.75 [0.10] m/s) (Figure 2A; eFigure 2 in the Supplement). In addition, the faces of slow-gaited adults were rated as looking older (β, −0.25; 95% CI, −0.31 to −0.18; *P* < .001) (Figure 2B).

### Gait Speed, Brain Structure, and Neurocognitive Functions at Age 45 Years

**Figure 3.**
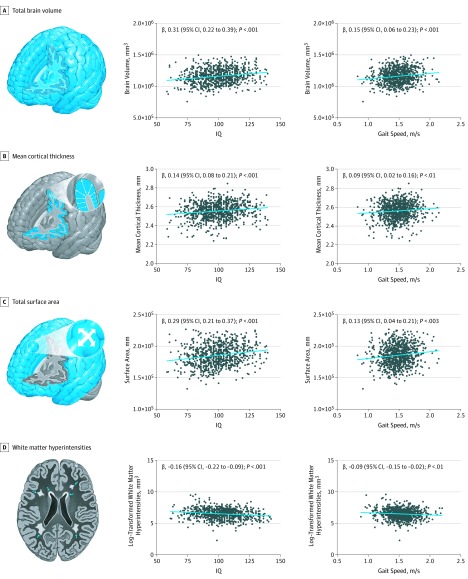
Associations Between Brain Structure With IQ and With Gait Speed at Age 45 Years Brain structure parameters shown include total brain volume (A), mean cortical thickness (B), total surface area (C), and log-transformed white matter hyperintensities (D). The left-hand scatterplots show associations between brain structure parameters and IQ at age 45 years. The right-hand scatterplots show associations between brain structure parameters and composite gait speed at age 45 years. β values represent standardized regression coefficients with 95% CIs adjusted for sex.

Midlife adults with smaller total brain volume (β, 0.15; 95% CI, 0.06 to 0.23; *P* < .001), thinner mean cortex (β, 0.09; 95% CI, 0.02 to 0.16; *P* = .01), smaller total brain surface area (β, 0.13; 95% CI, 0.04 to 0.21; *P* = .003), or a higher volume of white matter hyperintensities (β, −0.09; 95% CI, −0.15 to −0.02; *P* = .01) had slower gait (Table 2; Figure 3). The association between white matter hyperintensities and gait was not significant after controlling for body composition or childhood SES. These brain features (volume, cortical thickness, surface area, and white matter hyperintensity burden) are known to be associated with cognitive functioning, as confirmed by their associations with IQ (Figure 3). Next, we tested whether neurocognitive functioning at age 45 years was also associated with gait speed. Participants with lower IQ at age 45 years had slower gait (β, 0.38; 95% CI, 0.32 to 0.44; *P* < .001) (Table 2). The cognitive impairment of those with slow gait was apparent across multiple neuropsychological domains: they had slower processing speed (β, 0.30; 95% CI, 0.23 to 0.36; *P* < .001), poorer working memory (β, 0.32; 95% CI, 0.26 to 0.38; *P* < .001), poorer perceptual reasoning (β, 0.29; 95% CI, 0.22 to 0.35; *P* < .001), and poorer verbal comprehension (β, 0.30; 95% CI, 0.24 to 0.37) (Table 2). In addition, adults who performed worse on the Trail-Making Test (part A: β, 0.26; 95% CI, 0.20 to 0.33; part B: β, 0.22; 95% CI, 0.16 to 0.29; *P* < .001 for both), Animal Naming Test (β, 0.23; 95% CI, 0.16 to 0.29; *P* < .001), Wechsler Memory Scale–Mental Control (β, 0.20; 95% CI, 0.13 to 0.26; *P* < .001), and the Rey Auditory Verbal Learning Test of Memory (total, β, 0.33; 95% CI, 0.27 to 0.39; recall, β, 0.22; 95% CI, 0.15 to 0.29; *P* < .001 for both) had slower gait (Table 2).

### Childhood Neurocognitive Functioning and Gait Speed at Midlife

The contemporaneous association between gait speed and neurocognitive functioning at midlife was foreshadowed by cognitive differences already apparent in childhood (Figure 2C and D). We looked back to data obtained when participants were aged 3 years to test the longitudinal association between childhood brain health assessed during a pediatric examination and midlife gait. Indications of poor brain health at age 3 years were associated with slow gait at age 45 years (β, 0.26; 95% CI, 0.20-0.32; *P* < .001) (Figure 2C). Sensitivity analyses revealed that the following components of brain health at age 3 years were each significantly associated with gait speed: picture vocabulary, receptive language skills, motor skills, and lack of control (eTable 5 in the Supplement). The association between childhood brain health and adult gait speed held after controlling for childhood SES (β, 0.21; 95% CI, 0.15-0.28; *P* < .001). A decline on Wechsler testing from childhood to adulthood was associated with slower gait speed at midlife (β, 0.10; 95% CI, 0.04-0.17; *P* < .001) (Figure 2D), even after controlling for childhood SES (β, 0.11; 95% CI, 0.05-0.18; *P* < .001).

## Discussion

Gait speed is used primarily to monitor the functional capacity of older adults and to forecast their rate of age-related decline. Our findings suggest that gait speed may not only be a geriatric concern. In this 5-decade longitudinal study of a population-representative birth cohort, gait speed measured at age 45 years was already associated with physical and biological indicators of accelerated aging. Beyond accelerated aging, gait speed was associated with lifelong compromised brain health and neurocognitive functioning beginning as early as age 3 years. Notably, gait speed under the 3 separate walk conditions shared the same associations, albeit with larger effect sizes for the 2 challenge walks, especially maximum gait speed, which could suggest that maximum gait speed may be a more sensitive measure among midlife patients. These findings call for rethinking gait speed, from a geriatric index of functional decline to an index of the role of lifelong neurocognitive functioning in processes of aging. We next highlight 3 specific findings.

First, slow gait was associated with poor physical function at midlife. In this midlife cohort—most of whom walked faster than older adults^3^—we documented associations between gait speed and physical performance similar to those observed in older adults,^26^ indicating that the association between gait speed and physical function is evident at age 45 years, not just among older people.

Second, at midlife, slow gait was associated with accelerated aging, as indexed by more rapid deterioration of multiple organ systems over the preceding 2 decades and by facial age. Slow gait was also associated with multiple indices of compromised structural brain integrity, including smaller total brain volume, global cortical thinning, and reduced total surface area.^27^ Slow gait was associated with the volume of white matter hyperintensities, which is associated with cognitive decline and dementia,^28^ although this association diminished when we controlled for body composition or childhood SES. These findings in 45-year-old participants parallel those of studies^29,30^ showing that gait speed is associated with structural brain changes in older adults and that total brain volume and white matter hyperintensities are associated with gait slowing in older adults.^31,32^ These findings survived correction for multiple testing applied within the brain structure domain, but if correction for multiple testing had been applied across all study measures simultaneously, only total brain volume would have remained associated with gait speed, suggesting that findings for cortical thickness, surface area, and white matter hyperintensities, although consistent with the literature, should be treated with caution.

Third, slow gait at midlife was associated with poorer neurocognitive functioning across multiple cognitive domains; there was a mean difference of 16 IQ points (>1 SD) between the slowest and fastest walkers (ie, bottom vs top quintile). These findings align with those of several studies^4^ of older adults showing associations of slow gait with cognitive impairment and risk of dementia. Remarkably, in our study, gait speed was associated not only with concurrent neurocognitive functioning in adulthood but also with neurocognitive functioning in early childhood. The effect sizes between participants in the slowest and fastest gait speed quintiles were far from trivial: at age 3 years, the difference in brain health was 0.62 SD

Research is needed to unpack the association between childhood neurocognitive functioning and midlife gait speed. Six hypotheses are proposed: first, the link between better brain health and gait may be governed by the integrity of shared neural substrates that are involved in both neurocognitive functions and walking throughout life.^33,34^ Second, better brain health may be associated with health-promoting behaviors (eg, not smoking, healthy diet, and physical activity). Third, better brain health may be associated with better health literacy, facilitating access to better health care. Fourth, better brain health may be associated with higher education and lower risk of unsafe working conditions and health-damaging exposures. Fifth, better brain health is an early indicator of good overall physical status because the brain is a sensitive organ and possibly the first to indicate weak overall somatic system integrity across multiple organ systems.^35^ Sixth, common genetic factors may account for the link between better brain health and physical health, either because of lower mutation load^36^ or pleiotropy at genetic loci associated with both better neurocognitive function and a longer life span.^37^ The finding that midlife gait speed reflects lifelong compromised neurocognitive functioning may help to account for the robust ability of gait assessments to predict Alzheimer disease and related dementias.^4,32^

Because gait speed shows meaningful aging-related variation already in midlife, it may prove to be a useful measure in aging trials aimed at preventing the onset of age-related disease. A variety of interventions targeting human aging—ranging from calorie restriction to metformin administration^38,39^—are being tested in aging-prevention trials. It is increasingly recognized that it might be easier to prevent aging-associated damage than to reverse it, suggesting that the effect of interventions to slow aging may work better if they are applied while people are still young and free of disease and disability. This necessitates a shift toward enrolling younger participants in antiaging trials, and with this shift, valid measures are required to identify risk groups that need intervention, and to track the course of outcome before the manifestation of age-related diseases. Gait speed could be used as one such measure: the gait speed test is cheap, safe, easy to test repeatedly, and feasible to use among people in their 40s.

### Limitations

A limitation of the study is the lack of gait speed measurement before age 45 years, which precludes assessment of longitudinal changes in gait speed. Similarly, brain imaging data were not acquired before age 45 years. Although we were unable to examine structural brain changes over time, we were able to evaluate changes in neurocognitive functioning from childhood to adulthood and to show that greater cognitive decline from childhood to midlife was associated with slower midlife gait speed.

## Conclusions

Gait speed is more than just a geriatric index of adult functional decline; rather, it is a summary index of lifelong aging with possible origins in childhood CNS deficits. This helps to explain why gait can be such a powerful indicator of risk of disability and death in the elderly. It also encourages rethinking gait as not only a motoric concern, but as an integrative measure of health.

## Notes

Supplement. **eMethods 1.** Age 45 Assessment **eMethods 2.** Childhood Assessments **eMethods 3.** Attrition Analysis **eTable 1.** Associations Between Age-45 Measures of Physical Function, Accelerated Aging, Brain Structure, or Cognitive Function With Concurrent Composite Gait Speed in Models Adjusted for Leg Length and Body Composition (Lean Mass Index and Fat Mass Index) **eTable 2.** Associations Between Childhood Variables and Composite Gait Speed at Age 45 Years Adjusted for Leg Length and Body Composition (All Associations Are Adjusted for Sex and Childhood SES) **eTable 3.** Associations Between Age-45 Measures of Physical Function, Accelerated Aging, Brain Structure, or Cognitive Function With Concurrent Gait Speed (All Associations Are Adjusted for Sex and Childhood SES) **eTable 4.** Associations Between Childhood Variables and Gait Speed at Age 45 Years (All Associations Are Adjusted for Sex and Childhood SES) **eTable 5.** Associations Between Components of Childhood Brain Health and Composite Gait Speed at Age 45 Years **eFigure 1.** Distribution of Gait Speed for Participants in the Dunedin Study at Age 45 Years **eFigure 2.** Accelerated Aging and Poor Childhood Neurocognitive Functioning Are Associated With Slower Gait Speed at Age 45 Years **eReferences**
