# Supplementary material for: Association of Neurocognitive and Physical Function With Gait Speed in Midlife
Source: JAMA Netw Open. 2019 Oct 11;2(10):e1913123. doi: 10.1001/jamanetworkopen.2019.13123 (PMC6804027; doi:10.1001/jamanetworkopen.2019.13123)
Supplement: Supplement. — eMethods 1. Age 45 Assessment eMethods 2. Childhood Assessments eMethods 3. Attrition Analysis eTable 1. Associations Between Age-45 Measures of Physical Function, Accelerated Aging, Brain Structure, or Cognitive Function With Concurrent Composite Gait Speed in Models Adjusted for Leg Length and Body Composition (Lean Mass Index and Fat Mass Index) eTable 2. Associations Between Childhood Variables and Composite Gait Speed at Age 45 Years Adjusted for Leg Length and Body Composition (All Associations Are Adjusted for Sex and Childhood SES) eTable 3. Associations Between Age-45 Measures of Physical Function, Accelerated Aging, Brain Structure, or Cognitive Function With Concurrent Gait Speed (All Associations Are Adjusted for Sex and Childhood SES) eTable 4. Associations Between Childhood Variables and Gait Speed at Age 45 Years (All Associations Are Adjusted for Sex and Childhood SES) eTable 5. Associations Between Components of Childhood Brain Health and Composite Gait Speed at Age 45 Years eFigure 1. Distribution of Gait Speed for Participants in the Dunedin Study at Age 45 Years eFigure 2. Accelerated Aging and Poor Childhood Neurocognitive Functioning Are Associated With Slower Gait Speed at Age 45 Years eReferences [file jamanetwopen-2-e1913123-s001.pdf]

Supplementary Online Content

Rasmussen LJH, Caspi A, Ambler A, et al. Association of neurocognitive and physical function with gait speed in midlife. *JAMA Netw Open*. 2019;2(10):1913123. doi:10.1001/jamanetworkopen.2019.13123

eMethods 1. Age 45 Assessment

eMethods 2. Childhood Assessments

eMethods 3. Attrition Analysis

eTable 1. Associations Between Age-45 Measures of Physical Function, Accelerated Aging, Brain Structure, or Cognitive Function With Concurrent Composite Gait Speed in Models Adjusted for Leg Length and Body Composition (Lean Mass Index and Fat Mass Index)

eTable 2. Associations Between Childhood Variables and Composite Gait Speed at Age 45 Years Adjusted for Leg Length and Body Composition (All Associations Are Adjusted for Sex and Childhood SES)

eTable 3. Associations Between Age-45 Measures of Physical Function, Accelerated Aging, Brain Structure, or Cognitive Function With Concurrent Gait Speed (All Associations Are Adjusted for Sex and Childhood SES)

eTable 4. Associations Between Childhood Variables and Gait Speed at Age 45 Years (All Associations Are Adjusted for Sex and Childhood SES)

eTable 5. Associations Between Components of Childhood Brain Health and Composite Gait Speed at Age 45 Years

eFigure 1. Distribution of Gait Speed for Participants in the Dunedin Study at Age 45 Years

eFigure 2. Accelerated Aging and Poor Childhood Neurocognitive Functioning Are Associated With Slower Gait Speed at Age 45 Years

eReferences

This supplementary material has been provided by the authors to give readers additional information about their work.

© 2019 Rasmussen LJH et al. *JAMA Network Open.*

eMethods 1. Age 45 Assessment

Leg length and body composition (age 45)
Leg length was measured in cm from the top of iliac crest to the ground.

Body composition, including total body fat and lean mass, was measured by dual-energy X-ray absorptiometry (DXA) after removing any jewelry and metal objects. Scans were conducted using the automatic total body scan mode (GE Lunar Prodigy; Madison, WI, USA; 16.0 enCore software version with CoreScan™). Fat mass index was calculated as total body fat in kg divided by height in m^2. Lean mass index was calculated as total lean mass in kg divided by height in m^2. The scanner was calibrated daily with bone phantoms for quality assurance. The coefficients of variation (CVs) for repeat in vivo scans in adults for BMC (g) and area (g/cm^2), respectively, were: 1.8% for total fat mass and 1.0% for bone-free lean tissue mass.

Physical function (age 45)
Functional capacity at age 45 was assessed by self-reported physical limitations (RAND 36-Item Health Survey 1.0) and by several brief exercises that index the ability to perform everyday activities: handgrip strength, one-legged balance, visual-motor coordination, chair-stand test, and 2-min step test.

Physical limitations (RAND SF36)	Physical limitations were measured with the 10-item RAND 36-Item Health Survey 1.0 physical functioning scale.[1] Participant responses ("limited a lot", "limited a little", "not limited at all") assessed their difficulty with completing various activities, e.g., climbing several flights of stairs, walking more than 1 km, participating in strenuous sports, etc. Scores were reversed to reflect physical limitations so that a high score indicates more limitations.
Handgrip strength	Handgrip strength was measured for the dominant hand (elbow held at 90°, upper arm held tight against the trunk) as the maximum value achieved across three trials using a Jamar digital dynamometer.[2,3]
One-legged balance	Balance was measured using the Unipedal Stance Test as the maximum time achieved across three trials of the test with eyes closed.[4–6]
Visual-motor coordination	Visual-motor coordination was measured as the time to completion of the Grooved Pegboard Test with the non-dominant hand.[7] Scores for the Grooved Pegboard test were reversed so that higher values corresponded to better performance.
Chair-stand test	Chair rises were measured as the number of stands a participant completed in 30 seconds from a seated position.[8,9]
2-min step test	The 2-min step test was measured as the number of times a participant lifted their right knee to mid-thigh height (measured as the height half-way between the knee cap and the iliac crest) in 2 minutes at a self-directed pace.[9,10]

Pace of Aging (age 45)
Pace of Aging was measured for each Dunedin participant with repeated assessments of a panel of 19 biomarkers taken at ages 26, 32, 38, and 45 years; a method previously described.[11] The 19 biomarkers were: body mass index, waist-hip ratio, glycated hemoglobin (HbA1C), leptin, blood pressure (mean arterial pressure), cardiorespiratory fitness (VO_2Max), forced expiratory volume in one second (FEV_1), FEV_1 to forced vital capacity ratio (FEV_1/FVC), total cholesterol, triglycerides, high-density lipoprotein (HDL) cholesterol, apolipoprotein B100/A1 ratio, lipoprotein(a), creatinine clearance, urea nitrogen, C-reactive protein, white blood cell count, gum health, and caries-affected tooth surfaces. Measures were taken in counterbalanced order across participants with the exception of blood, which was drawn at the same time of day for all participants at all four ages and dental examinations which were conducted in the late afternoon at all four ages. Women who were pregnant at the time of a given assessment were excluded from that wave of data collection. The measurement of each biomarker is described below. Change over time in each biomarker was modeled with mixed-effects growth models, and these rates of change were combined into a single index scaled (within sex) in years of physiological change occurring per one chronological

© 2019 Rasmussen LJH et al. *JAMA Network Open.*

year, as per the method previously described.[11] Participants ranged in their Pace of Aging from near 0 years of physiological change per chronological year to nearly 3 years of physiological change per chronological year.

Body mass index	Height was measured to the nearest millimeter using a portable stadiometer (Harpenden; Holtain, Ltd.). Weight was measured to the nearest 0.1 kg using calibrated scales. Individuals were weighed in light clothing. Body mass index (BMI) was calculated.
Waist-hip ratio	Waist girth was the perimeter at the level of the noticeable waist narrowing located between the costal border and the iliac crest. Hip girth was taken as the perimeter at the level of the greatest protuberance and at about the symphysion pubic level anteriorly. Measurements were repeated and the average used to calculate waist-hip ratio.
Glycated hemoglobin (HbA1C)	Whole blood glycated hemoglobin concentration (expressed as a percentage of total hemoglobin) was measured by ion exchange high performance liquid chromatography (Variant II: BioRad, Hercultes, Calif.), a method certified by the US National Glycohemoglobin Standardization Program (http://www.ngsp.org/).
Leptin	Serum leptin (μg/L) was measured using the Quantikine ELISA Human Leptin Immunoassay (Cat# SLP00, R&D Systems Inc, Minneapolis, MN) according to the manufacturer's instruction.
Blood pressure (mean arterial pressure)	Systolic and diastolic blood pressure were assessed according to standard protocols with a Hawksley random-zero sphygmomanometer with a constant deflation valve. Mean arterial pressure (MAP) was calculated using the formula Diastolic Pressure+1/3(Systolic Pressure - Diastolic Pressure).
Cardiorespiratory fitness (VO_2Max)	Cardiorespiratory fitness was assessed by measuring heart rate in response to a submaximal exercise test on a friction-braked cycle ergometer. Dependent on the extent to which heart rate increased during a 2-min 50 W warm-up, the workload was adjusted to elicit a steady heart-rate in the range 130–170 beats per minute. After a further 6-min constant power output stage, the maximum heart rate was recorded and used to calculate predicted maximum oxygen uptake adjusted for body weight in milliliters per minute per kilogram (VO_2max) according to standard protocols.[12]
Lung function (FEV_1 and FEV_1/FVC)	We calculated post-albuterol forced expiratory volume in one second (FEV_1) and the ratio of FEV_1 to forced vital capacity (FVC; FEV_1/FVC) using measurements from spirometry conducted with a Sensormedics body plethysmograph (Sensormedics Corporation, Yorba Linda, CA, USA).
Total cholesterol, triglycerides, and high-density lipoprotein (HDL) cholesterol	Serum non-fasting total cholesterol, triglycerides, and high-density lipoprotein (HDL) cholesterol levels (mmol/L) were measured by colorimetric assay on a Hitachi 917 analyzer (ages 26-32), a Modular P analyzer (age 38), and a Cobas c702 analyzer (age 45).
Apolipoprotein B100/A1 ratio	Serum apolipoprotein A1 and apolipoprotein B100 (g/L) were measured by immunoturbidimetric assay on a Hitachi 917 analyzer (ages 26-32), a Modular P analyzer (age 38), and a Cobas c502 (age 45), and the ratio between the two was calculated.
Lipoprotein(a)	Serum lipoprotein(a) (nmol/L) was measured by a particle-enhanced immunoturbidimetric assay on a Hitachi 917 analyzer (ages 26-32), a Modular P analyzer (age 38), and a Cobas c502 analyzer (age 45).

© 2019 Rasmussen LJH et al. *JAMA Network Open.*

Creatinine clearance	Serum creatinine (mmol/L) was measured by kinetic colorimetric assay on a Hitachi 917 analyzer (age 32), Modular P analyzer (age 38), and Cobas c702 (age 45) (Roche Diagnostics, Mannheim, Germany). For Pace of Aging analysis, creatinine was measured as creatinine clearance, calculated using the National Kidney Foundation CKD-EPI Creatinine Equation (2009).[13]
Urea nitrogen	Serum urea nitrogen (mmol/L) was measured by kinetic UV assay at ages 26 (Hitachi 917 analyzer) and 45 (Cobas c702 analyzer), and by kinetic colorimetric assay at ages 32 (Hitachi 917 analyzer) and 38 (Modular P analyzer).
High sensitivity C-reactive protein (hsCRP)	Serum C-reactive protein (mg/L) was measured by high sensitivity immunoturbidimetric assay on a Hitachi 917 analyzer (age 32), a Modular P analyzer (age 38), and a Cobas c702 (age 45). Values were log-transformed for analysis.
White blood cell count	Whole blood white blood cell counts ($x10^9$/L) were measured by flow cytometry with a Coulter STKS (Coulter Corporation, Miami, FL) (age 26), a Sysmex XE2100 (Sysmex Corporation, Japan) (age 32), and a Sysmex XE5000 (Sysmex Corporation, Japan) (ages 38 and 45). Counts were log-transformed for analysis.
Gum health (combined attachment loss)	Calibrated dentists examined periodontal health at three sites (mesiobuccal, buccal, and distolingual) per tooth. Gingival recession (the distance in millimeters from the cementoenamel junction to the gingival margin) and probing depth (the distance from the probe tip to the gingival margin) were recorded using a PCP-2 periodontal probe (Hu-Friedy; Chicago). The combined attachment loss for each site was computed by summing gingival recession and probing depth (third molars were not included) and then averaged across all periodontally examined teeth.
Caries-affected tooth surfaces	Teeth were examined for caries and restorations following the World Health Organization Oral Health Surveys methodology.[14,15] Four surfaces were considered for anterior teeth (canines and incisors): buccal, lingual, distal, and mesial; a fifth surface, occlusal, was considered for premolar and molar teeth. Tooth surfaces were classified as having untreated caries (DS) if a cavitated carious lesion was present, as filled (FS) if a dental restoration was present (including crowns), and missing due to caries (MS) if the participant indicated that a given tooth had been removed due to decay or failed dental restorative work. DS, MS, and FS counts were summed (ranging from 0 to 148 surfaces). Surfaces of teeth that were unerupted, lost due to trauma, extracted for reasons other than caries (impaction, orthodontic treatment, or periodontal disease), or could not be visualized by the examiner were excluded from calculations.

Facial Aging (age 45)
Facial Aging was based on ratings by an independent panel of 8 raters of each participant's facial photograph. Facial Aging was based on two measurements of perceived age. First, Age Range was assessed by an independent panel of 4 raters, who were presented with standardized (non-smiling) facial photographs of participants and were kept blind to their actual age. Raters used a Likert scale to categorize each participant into a 5-year age range (i.e., from 20-24 years old up to 70+ years old) (interrater reliability = .77). Scores for each participant were averaged across all raters. Second, Relative Age was assessed by a different panel of 4 raters, who were told that all photos were of people aged 45 years old. Raters then used a 7-item Likert scale to assign a "relative age" to each participant (1="young looking", 7="old looking") (interrater reliability = .79). The measure of perceived age at 45 years, Facial Age, was derived by standardizing and averaging Age Range and Relative Age scores.

Structural MRI (age 45)
Image Acquisition. Each participant was scanned using a Siemens Skyra 3T scanner equipped with a 64-channel head/neck coil at the Pacific Radiology imaging center in Dunedin, New Zealand. High resolution T1-weighted

images were obtained using an MP-RAGE sequence with the following parameters: TR = 2400 ms; TE = 1.98 ms; 208 sagittal slices; flip angle, 9°; FOV, 224 mm; matrix =256×256; slice thickness = 0.9 mm with no gap (voxel size 0.9×0.875×0.875 mm); and total scan time = 6 min and 52 s. 3D fluid-attenuated inversion recovery (FLAIR) images were obtained with the following parameters: TR = 8000 ms; TE = 399 ms; 160 sagittal slices; FOV = 240 mm; matrix = 232×256; slice thickness = 1.2 mm (voxel size 0.9×0.9×1.2 mm); and total scan time = 5 min and 38 s. Additionally, a gradient echo field map was acquired with the following parameters: TR = 712 ms; TE = 4.92 and 7.38 ms; 72 axial slices; FOV = 200 mm; matrix = 100×100; slice thickness = 2.0 mm (voxel size 2 mm isotropic); and total scan time = 2 min and 25 s.

Image Processing. Structural MRI data were analyzed using the Human Connectome Project (HCP) minimal preprocessing pipeline as extensively detailed elsewhere.[16] Briefly, T1-weighted and FLAIR images were processed through the PreFreeSurfer, FreeSurfer, and PostFreeSurfer pipelines. T1-weighted and FLAIR images were corrected for readout distortion using the gradient echo field map, coregistered, brain-extracted, and aligned together in the native T1 space using boundary-based registration.[17] Images were then processed with a custom FreeSurfer recon-all pipeline that is optimized for structural MRI with higher resolution than 1 mm isotropic. Finally, recon-all output were converted into CIFTI format and registered to common 32k_FS_LR mesh using MSM-sulc.[18] Outputs of the minimal preprocessing pipeline were visually checked for accurate surface generation by examining each participant's myelin map, pial surface, and white matter boundaries.

For brain volume, cortical thickness, and surface area data, participants were excluded if they failed visual inspection of surface generation, had major incidental findings, or were missing T2-weighted or field map scans, yielding 861 datasets for analyses.

Total brain volume

Total brain volume was extracted from the FreeSurfer "aseg" parcellation.

Cortical thickness and surface area

For each participant the mean cortical thickness and surface area were then extracted from each of the 360 cortical areas in the HCP-MPP1.0 parcellation.[19]

White matter hyperintensities

To identify and extract the total volume of white matter hyperintensities (WMHs), T1-weighted and FLAIR images for each participant were processed with the UBO Detector, a cluster-based, fully-automated pipeline with high reliability in our data (test-retest ICC = 0.87) and out of sample performance.[20] The resulting WMH probability maps were thresholded at 0.7, which is the suggested standard. WMH volume is measured in Montreal Neurological Institute (MNI) space, thus removing the influence of differences in brain volume on WMH volume. Because of the potential for bias and false positives due to the thresholds and masks applied in UBO, the resulting WMH maps for each participant were manually checked by two independent raters to ensure that false detections did not substantially contribute to estimates of WMH volume. Visual inspections were done blind to the participants' cognitive status. Due to the tendency of automated algorithms to mislabel regions surrounding the septum as WMHs, these regions were manually masked out, to further ensure the most accurate grading possible. For WMH data, participants were excluded if they had missing FLAIR scans, Multiple Sclerosis, or inaccurate white matter labelling or low-quality MRI data, yielding 852 datasets for analyses. In all analyses, WMH volume was log-transformed.

Cognitive functioning (age 45)

The Wechsler Adult Intelligence Scale-IV (WAIS-IV)[21] was administered to the participants at age 45 years, yielding the IQ. In addition to full scale IQ, the WAIS-IV measures four specific domains of cognitive function: Processing Speed, Working Memory, Perceptual Reasoning, and Verbal Comprehension. Participants were also tested with an additional suite of measures of executive functioning, verbal fluency, and memory: the Trail-Making Test, Animal Naming Test, Wechsler Memory Scale – Mental Control, and the Rey Auditory Verbal Learning Test. The tests were individually administered according to standard protocol. Examiners were blind to the participants' performance on previous administration of the tests. WAIS-IV subtests are the same as for the corresponding WISC-R subtests, but with appropriate item difficulty for adults, allowing the study of cognitive decline. Details about the Trail-Making Test, Animal Naming Test, Wechsler Memory Scale – Mental Control, and the Rey Auditory Verbal Learning Test are provided below.

© 2019 Rasmussen LJH et al. *JAMA Network Open.*

Trail-Making Test	The Trail Making Test[22] (for adults) is a non-verbal test of executive functioning and consists of two separate tests. Part A has been suggested to be a measure of visual search and motor speed skills. Part B is additionally a measure of task-switching, mental flexibility, and related higher order cognitive skills. Participants were given standard instructions and a demonstration of both Parts A and B of the test.
Animal Naming Test	The Animal Naming Test[23] is a test of memory and verbal fluency. Participants were asked to name as many animals as they can think of, as quickly as possible in 60 seconds. The total score was the number of animals, excluding errors of intrusion or perseveration. Credit can be given for all animals, including birds, fish, reptiles, insects, humans, extinct animals, etc., for general category terms (e.g., dog) and for specific instances (e.g., terriers) when both are given. Only one item is credited if the same animal at different developmental stages (e.g., sheep, lamb) is named.
Wechsler Memory Scale – Mental Control	The Wechsler Memory Scale (WMS-III)[24] test of Mental Control is a test of attention and tracking. It requires reciting the months of the year in backwards order, starting with December. Responses were scored according to the instructions in the WMS-III manual. Scores ranged from 1 (poor performance) to 5 (good performance) and reflect both accuracy and speed.
Rey Auditory Verbal Learning Test	This is a test of verbal learning and memory administered at 45 years.[7] The test involves a five-trial presentation of a 15-word list and a one-time presentation of an interference list. Four trials of the 15-word list were administered due to time constraints. Words are recalled immediately after each trial and later after a 25-30 minute delay. Total Recall is the total number of words (0-60) recalled over four trials (the sum of words recalled across trials 1-4). Delayed Recall is the total number of words (0-15) recalled after a 25-30 minute delay.

© 2019 Rasmussen LJH et al. *JAMA Network Open.*

eMethods 2. Childhood Assessments

Childhood Brain Health (age 3)
Brain Health was assessed with a summary factor score indexing the child's early neurocognitive status based on information from the child examination and behavior ratings. At age 3 years, each child participated in a 45-minute examination that included assessment by a pediatric neurologist and standardized tests of intelligence, receptive language, and motor skills. Afterwards the examiners (having no prior knowledge of the child) rated each child's emotional and behavioral regulation. The summary score was created with confirmatory factor analysis of these five indicators of Brain Health assessed at age 3: neurological abnormalities, lack of control, receptive language, Peabody Picture Vocabulary Test, and motor development[25]. The model fit the data well, $\chi 2$ (N = 1,035, df = 5) = 6.459, p = .2641, CFI = .999, TLI = .997, RMSEA = 0.17. Factor scores were output and standardized to a mean = 0 and SD = 1.

Neurological abnormalities	At age 3 years, each child was examined by a pediatric neurologist for neurological signs, including assessment of motility, passive movements, reflexes, facial musculature, strabismus, nystagmus, foot posture, and gait, based on procedures described by Touwen and Prechtl (1970).[26]
Peabody Picture Vocabulary test	Intelligence was assessed at age 3 years with the Peabody Picture Vocabulary Test.[27]
Receptive language	Receptive language was assessed at age 3 years with Reynell Developmental Language Scales.[28]
Motor development	Motor development was assessed at age 3 years with the Bayley Motor Scales.[29]
Lack of control	At age 3 years, each child participated in a testing session involving cognitive and motor tasks. The children were tested by examiners who had no knowledge of their behavioral history. Following the testing, each examiner rated the child's lack of control in the testing session, yielding a behavioral style factor, labeled Lack of Control,[30] which characterized children who were labile, had low frustration tolerance, lacked reserve, were resistant, restless, impulsive, required attention, and lacked persistence in reaching goals.

Wechsler Intelligence Scale for Children–Revised
The Wechsler Intelligence Scale for Children–Revised (WISC–R)[31] was administered to the participants at ages 7, 9, and 11 years, yielding the IQ. Scores were averaged across the three assessments. The tests were individually administered at each assessment according to standard protocol. Examiners were blind to the children's performance on previous administrations of the WISC–R.

Childhood socioeconomic status
The socioeconomic statuses of cohort members' childhood families were measured using a 6-point Elley-Irving socioeconomic index for New Zealand[32] that assessed parents' occupational statuses, defined based on average income and educational levels derived from the New Zealand Census. The highest occupational status of either parent was averaged across the childhood assessments at ages 3, 5, 7, 9, 11, 13, and 15 years.

© 2019 Rasmussen LJH et al. *JAMA Network Open.*

eMethods 3. Attrition Analysis

We conducted an attrition analysis using childhood neurocognitive functioning (the Wechsler Intelligence Scale for Children–Revised; WISC–R) and socioeconomic status (SES) to determine whether participants in the Phase 45 data collection were representative of the original cohort.

No significant differences in WISC–R were found between the full cohort, those still alive, those seen at Phase 45, or those with gait data at Phase 45. Those who were deceased by the Phase 45 data collection had significantly lower scores on the WISC–R than those who were still alive (t=2.09, *P*=.04).

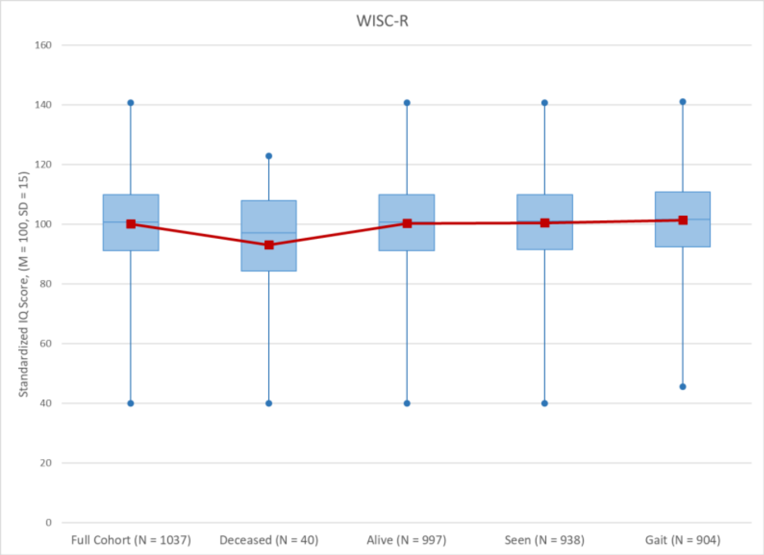

No significant differences were found between the full cohort, those deceased, those alive, those seen at Phase 45, or those with gait data at Phase 45 on childhood SES.

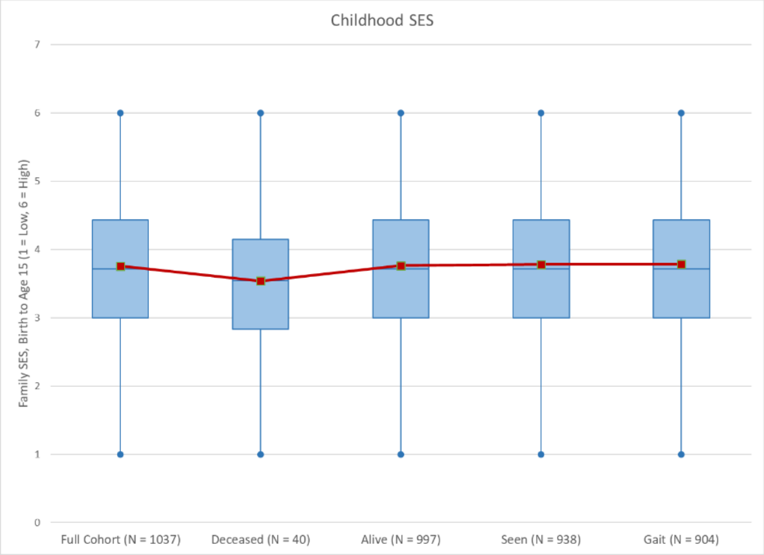

© 2019 Rasmussen LJH et al. *JAMA Network Open.*

eTable 1. Associations Between Age-45 Measures of Physical Function, Accelerated Aging, Brain Structure, or Cognitive Function With Concurrent Composite Gait Speed in Models Adjusted for Leg Length and Body Composition (Lean Mass Index and Fat Mass Index)[a]

	Adjusted for leg length			Adjusted for lean mass and fat mass			Adjusted for leg length, lean mass, and fat mass		
**Variable**	**n**	***β* (95% CI)**	***P***	**n**	***β* (95% CI)**	***P***	**n**	***β* (95% CI)**	***P***
**Functional capacity**									
Physical limitations[b]	901	-0.27 (-0.33 to -0.21)	<.001	892	-0.20 (-0.27 to -0.14)	<.001	892	-0.21 (-0.27 to -0.14)	<.001
Maximum handgrip strength, kg	903	0.28 (0.17 to 0.39)	<.001	894	0.33 (0.23 to 0.43)	<.001	894	0.26 (0.15 to 0.37)	<.001
One-legged balance, s	897	0.27 (0.21 to 0.33)	<.001	888	0.20 (0.13 to 0.26)	<.001	888	0.19 (0.13 to 0.26)	<.001
Visual-motor coordination[c]	899	0.23 (0.16 to 0.29)	<.001	890	0.22 (0.15 to 0.28)	<.001	890	0.21 (0.15 to 0.27)	<.001
Chair-stand test, no. in 30 s	873	0.35 (0.28 to 0.40)	<.001	866	0.28 (0.21 to 0.34)	<.001	866	0.29 (0.22 to 0.35)	<.001
2-min step test, no.	886	0.32 (0.25 to 0.38)	<.001	878	0.29 (0.22 to 0.34)	<.001	878	0.27 (0.21 to 0.33)	<.001
**Accelerated aging**									
Pace of Aging[d]	903	-0.31 (-0.37 to -0.25)	<.001	894	-0.23 (-0.31 to -0.16)	<.001	894	-0.21 (-0.29 to -0.14)	<.001
Facial Aging	902	-0.23 (-0.29 to -0.17)	<.001	894	-0.21 (-0.27 to -0.15)	<.001	894	-0.20 (-0.26 to -0.14)	<.001
**Brain structure**									
Total brain volume, mm^3	859	0.11 (0.02 to 0.19)	.01	852	0.14 (0.06 to 0.22)	.001	852	0.10 (0.02 to 0.18)	.02
Mean cortical thickness, mm	859	0.09 (0.02 to 0.15)	.01	852	0.08 (0.01 to 0.14)	.02	852	0.08 (0.01 to 0.14)	.02
Total surface area, mm^2	859	0.08 (-0.001 to 0.17)	.05	852	0.12 (0.04 to 0.20)	.003	852	0.08 (0.003 to 0.16)	.04
Total log(WMH), mm^3	849	-0.08 (-0.14 to -0.01)	.03	842	-0.06 (-0.12 to 0.01)	.08	842	-0.05 (-0.11 to 0.01)	.13
**Cognitive function**									
Total IQ	902	0.36 (0.30 to 0.42)	<.001	893	0.35 (0.29 to 0.41)	<.001	893	0.33 (0.27 to 0.39)	<.001
Processing Speed	902	0.28 (0.22 to 0.34)	<.001	893	0.28 (0.22 to 0.34)	<.001	893	0.26 (0.20 to 0.32)	<.001
Working Memory	898	0.30 (0.24 to 0.37)	<.001	889	0.29 (0.23 to 0.35)	<.001	889	0.27 (0.22 to 0.34)	<.001
Perceptual Reasoning	902	0.27 (0.20 to 0.33)	<.001	893	0.26 (0.20 to 0.32)	<.001	893	0.24 (0.18 to 0.30)	<.001
Verbal Comprehension	892	0.28 (0.22 to 0.34)	<.001	883	0.28 (0.22 to 0.34)	<.001	883	0.26 (0.20 to 0.32)	<.001
Trail-Making Test A[e]	901	0.25 (0.19 to 0.31)	<.001	892	0.26 (0.20 to 0.32)	<.001	892	0.25 (0.19 to 0.31)	<.001
Trail-Making Test B[e]	902	0.21 (0.14 to 0.27)	<.001	893	0.21 (0.15 to 0.27)	<.001	893	0.19 (0.13 to 0.25)	<.001

© 2019 Rasmussen LJH et al. *JAMA Network Open.*

Animal Naming Test	895	0.22 (0.15 to 0.28)	<.001	886	0.20 (0.14 to 0.26)	<.001	886	0.19 (0.13 to 0.25)	<.001
Wechsler Memory Scale–Mental Control[f]	888	0.18 (0.12 to 0.25)	<.001	880	0.19 (0.12 to 0.25)	<.001	880	0.18 (0.11 to 0.24)	<.001
RAVL test (Total)	902	0.31 (0.25 to 0.38)	<.001	893	0.29 (0.23 to 0.36)	<.001	893	0.28 (0.22 to 0.34)	<.001
RAVL test (Recall)	898	0.20 (0.14 to 0.27)	<.001	889	0.20 (0.13 to 0.26)	<.001	889	0.18 (0.12 to 0.25)	<.001

Abbreviations: CI, confidence interval; RAVL, Rey Auditory Verbal Learning; WMH, white matter hyperintensities.

[a] Standardized regression (β) coefficients. All analyses are sex-adjusted.

[b] RAND 36-Item Health Survey 1.0 physical functioning scale; reversed scores to reflect limitations.

[c] Grooved pegboard test, time (s) for non-dominant hand. Scores were reversed so that higher values corresponded to better performance.

[d] Years of physiological change per chronological year.

[e] Scores for the Trail-Making Tests were reversed so that higher values corresponded to better cognitive performance.

[f] Naming the months backward.

eTable 2. Associations Between Childhood Variables and Composite Gait Speed at Age 45 Years Adjusted for Leg Length and Body Composition (All Associations Are Adjusted for Sex and Childhood SES)[a]

	Adjusted for leg length			Adjusted for lean mass and fat mass			Adjusted for leg length, lean mass, and fat mass		
**Variable**	**n**	***β* (95% CI)**	***P***	**n**	***β* (95% CI)**	***P***	**n**	***β* (95% CI)**	***P***
Child-to-adult cognitive decline	888	0.11 (0.05–0.18)	<.001	879	0.11 (0.05–0.18)	<.001	879	0.11 (0.05–0.18)	<.001
Childhood Brain Health	897	0.19 (0.13–0.26)	<.001	888	0.21 (0.14–0.27)	<.001	888	0.19 (0.13–0.25)	<.001

Abbreviations: CI, confidence interval; SES, socioeconomic status.

[a] Standardized regression (β) coefficients. All analyses are adjusted for sex and childhood SES.

© 2019 Rasmussen LJH et al. *JAMA Network Open.*

eTable 3. Associations Between Age-45 Measures of Physical Function, Accelerated Aging, Brain Structure, or Cognitive Function With Concurrent Gait Speed (All Associations Are Adjusted for Sex and Childhood SES)[a]

		Usual gait speed		Dual task gait speed		Maximum gait speed		Composite gait speed	
**Variable**	**n**	***β* (95% CI)**	***P***	***β* (95% CI)**	***P***	***β* (95% CI)**	***P***	***β* (95% CI)**	***P***
**Physical function**									
Physical limitations[b]	896	-.19 (-.25; -.12)	<.001	-.17 (-.24; -.11)	<.001	-.25 (-.31; -.19)	<.001	-.25 (-.31; -.19)	<.001
Maximum handgrip strength, kg	898	.15 (.05; .26)	.005	.23 (.12; .34)	<.001	.39 (.29; .49)	<.001	.34 (.23; .44)	<.001
One-legged balance, s	892	.14 (.07; .20)	<.001	.17 (.11; .24)	<.001	.28 (.21; .34)	<.001	.25 (.19; .31)	<.001
Visual-motor coordination[c]	894	.10 (.03; .17)	.003	.19 (.13; .26)	<.001	.21 (.15; .27)	<.001	.21 (.15; .28)	<.001
Chair stands, no. in 30 s	868	.19 (.12; .25)	<.001	.23 (.16; .29)	<.001	.34 (.27; .40)	<.001	.32 (.25; .38)	<.001
2-min step test, no.	881	.15 (.08; .21)	<.001	.20 (.14; .27)	<.001	.36 (.30; .42)	<.001	.31 (.24; .37)	<.001
**Accelerated aging**									
Pace of Aging[d]	898	-.23 (-.30; -.17)	<.001	-.25 (-.31; -.18)	<.001	-.27 (-.33; -.21)	<.001	-.30 (-.37; -.24)	<.001
Facial Aging	897	-.14 (-.21; -.08)	<.001	-.15 (-.21; -.08)	<.001	-.22 (-.28; -.15)	<.001	-.21 (-.28; -.15)	<.001
**Brain structure**									
Total brain volume, mm^3	854	.07 (-.01; .16)	.09	.11 (.03; .20)	.009	.10 (.01; .18)	.02	.12 (.03; .20)	.006
Mean cortical thickness, mm	854	.05 (-.02; .11)	.15	.06 (-.003; .13)	.06	.06 (-.01; .13)	.07	.07 (.005; .14)	.04
Total surface area, mm^2	854	.07 (-.01; .15)	.10	.09 (.01; .18)	.03	.07 (-.01; .15)	.10	.09 (.01; .18)	.03
Total log(WMH), mm^3	844	-.03 (-.10; .04)	.37	-.07 (-.14; -.004)	.04	-.06 (-.12; .01)	.09	-.07 (-.13; -.001)	.05
**Cognitive function**									
Total IQ	897	.19 (.12; .26)	<.001	.28 (.21; .35)	<.001	.37 (.30; .43)	<.001	.35 (.29; .42)	<.001
Processing speed	897	.15 (.09; .22)	<.001	.25 (.19; .32)	<.001	.23 (.17; .30)	<.001	.27 (.20; .33)	<.001
Working memory	893	.17 (.10; .24)	<.001	.24 (.17; .31)	<.001	.28 (.22; .35)	<.001	.29 (.22; .35)	<.001
Perceptual reasoning	897	.10 (.04; .17)	.003	.19 (.12; .25)	<.001	.29 (.23; .35)	<.001	.25 (.19; .32)	<.001
Verbal comprehension	887	.14 (.07; .21)	<.001	.17 (.10; .24)	<.001	.31 (.24; .37)	<.001	.26 (.19; .33)	<.001
Trail-Making Test A[e]	896	.14 (.07; .20)	<.001	.22 (.15; .28)	<.001	.22 (.16; .28)	<.001	.24 (.18; .30)	<.001
Trail-Making Test B[e]	897	.08 (.02; .15)	.01	.16 (.09; .23)	<.001	.20 (.14; .27)	<.001	.19 (.13; .26)	<.001
Animal Naming, no. in 60 s	890	.11 (.05; .18)	.001	.14 (.07; .21)	<.001	.21 (.15; .28)	<.001	.20 (.13; .26)	<.001

© 2019 Rasmussen LJH et al. *JAMA Network Open.*

Wechsler Memory Scale–Mental Control[f]	883	.11 (.04; .18)	.001	.12 (.05; .18)	<.001	.16 (.09; .22)	<.001	.16 (.09; .23)	<.001
RAVL test (Total)	897	.17 (.10; .24)	<.001	.22 (.15; .29)	<.001	.30 (.24; .37)	<.001	.29 (.23; .36)	<.001
RAVL test (Recall)	893	.11 (.04; .18)	.002	.13 (.06; .20)	<.001	.19 (.13; .26)	<.001	.18 (.12; .25)	<.001

Abbreviations: CI, confidence interval; RAVL, Rey Auditory Verbal Learning; SD, standard deviation; SES, socioeconomic status; WMH, white matter hyperintensities.

[a] Sex- and SES-adjusted standardized regression (β) coefficients.

[b] RAND 36-Item Health Survey 1.0 physical functioning scale; reversed scores to reflect limitations

[c] Grooved pegboard test, time (s) for non-dominant hand. For the correlation analyses, scores were reversed so that higher values corresponded to better performance.

[d] Years of physiological change per chronological year.

[e] Scores for the Trail-Making Tests were reversed so that higher values corresponded to better cognitive performance.

[f] Naming the months backward.

eTable 4. Associations Between Childhood Variables and Gait Speed at Age 45 Years (All Associations Are Adjusted for Sex and Childhood SES)[a]

		Usual gait speed		Dual task gait speed		Maximum gait speed		Composite gait speed	
**Variable**	**No.**	***β* (95% CI)**	***P***	***β* (95% CI)**	***P***	***β* (95% CI)**	***P***	***β* (95% CI)**	***P***
Child-to-adult cognitive decline	888	.08 (.01; .14)	.02	.12 (.06; .19)	<.001	.08 (.02; .14)	.02	.11 (.05; .18)	<.001
Childhood Brain Health	897	.08 (.02; .15)	.01	.16 (.09; .23)	<.001	.25 (.18; .31)	<.001	.21 (.15; .28)	<.001

Abbreviations: CI, confidence interval; SES, socioeconomic status.

[a] Sex- and SES-adjusted standardized regression (β) coefficients.

© 2019 Rasmussen LJH et al. *JAMA Network Open.*

eTable 5. Associations Between Components of Childhood Brain Health and Composite Gait Speed at Age 45 Years[a]

**Variable**	**n**	***β*** **(95% CI)**	***P*** **value**
Neurological Abnormalities	854	-0.05 (-0.12 to 0.02)	.13
Peabody Picture Vocabulary Test	858	0.23 (0.16 to 0.29)	<.001
Receptive Language	896	0.24 (0.17 to 0.30)	<.001
Bayley Motor Scales	851	0.10 (0.04 to 0.17)	.002
Lack of Control	894	-0.15 (-0.21 to -0.09)	<.001

Abbreviations: CI, confidence interval.

[a] Sex-adjusted standardized regression (*β*) coefficients.

© 2019 Rasmussen LJH et al. *JAMA Network Open.*

eFigure 1. Distribution of Gait Speed for Participants in the Dunedin Study at Age 45 Years

Gait speed distributions for individual walk conditions (usual, dual task, maximum) and Composite gait speed are depicted as histograms. Scatterplots illustrate the pairwise correlations between individual walk conditions and Composite gait speed. The blue lines are linear regression lines with the shaded areas indicating 95% confidence intervals.

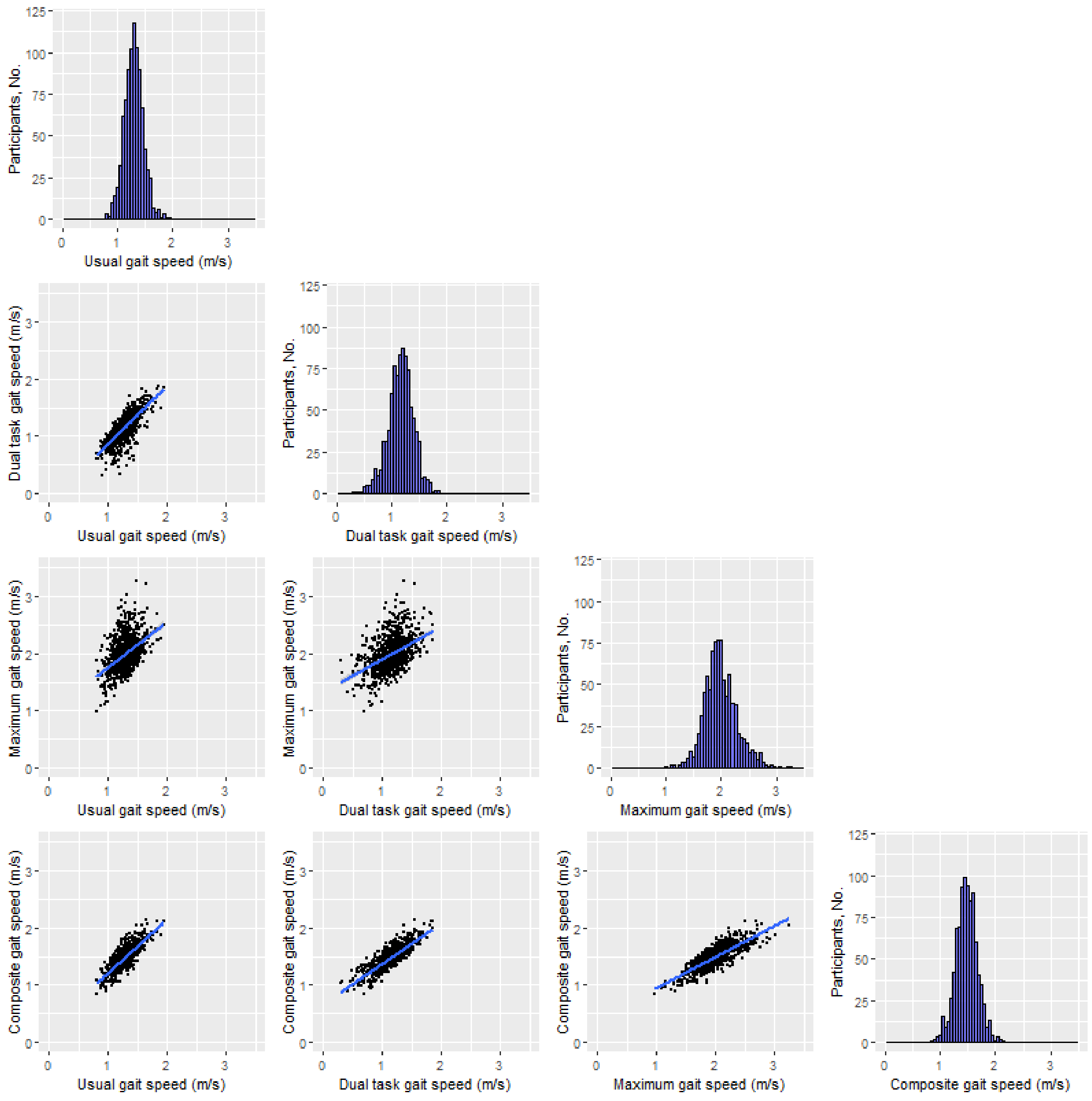

© 2019 Rasmussen LJH et al. *JAMA Network Open.*

eFigure 2. Accelerated Aging and Poor Childhood Neurocognitive Functioning Are Associated With Slower Gait Speed at Age 45 Years

Generalized additive models of Pace of Aging (age 45), Facial Aging (age 45), and childhood Brain Health (age 3) by Composite gait speed at age 45. Models were fitted with df=6, using the gam() package in R.

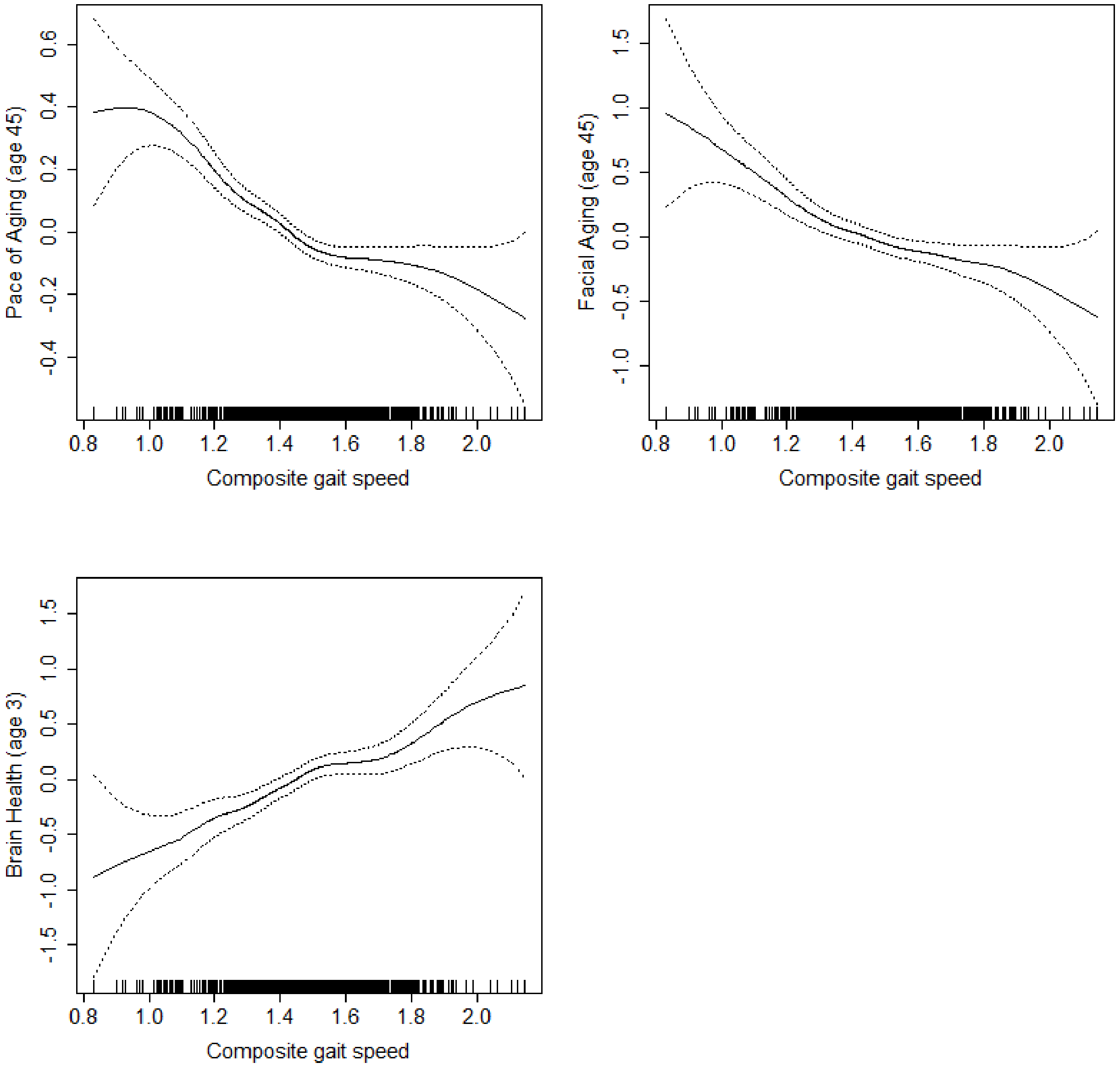

© 2019 Rasmussen LJH et al. *JAMA Network Open.*

eReferences

1. RAND 36-Item Short Form Survey (SF-36). https://www.rand.org/health-care/surveys_tools/mos/36-item-short-form.html. Accessed August 14, 2019.
2. Rantanen T, Guralnik JM, Foley D, et al. Midlife hand grip strength as a predictor of old age disability. *JAMA*. 1999;281(6):558-560.
3. Mathiowetz V, Kashman N, Volland G, Weber K, Dowe M, Rogers S. Grip and pinch strength: normative data for adults. *Arch Phys Med Rehabil*. 1985;66(2):69-74.
4. Bohannon RW, Larkin PA, Cook AC, Gear J, Singer J. Decrease in timed balance test scores with aging. *Phys Ther*. 1984;64(7):1067-1070.
5. Vereeck L, Wuyts F, Truijen S, Van de Heyning P. Clinical assessment of balance: Normative data, and gender and age effects. *Int J Audiol*. 2008;47(2):67-75.
6. Springer BA, Marin R, Cyhan T, Roberts H, Gill NW. Normative values for the unipedal stance test with eyes open and closed. *J Geriatr Phys Ther*. 2007;30(1):8-15.
7. Lezak D, Howieson D, Loring D, Hannay H, Fischer J. *Neuropsychological Assessment, 4th Edition*. New York: Oxford Univ Press, New York; 2004.
8. Jones CJ, Rikli RE, Beam WC. A 30-s Chair-Stand Test as a Measure of Lower Body Strength in Community-Residing Older Adults. *Res Q Exerc Sport*. 1999;70(2):113-119.
9. Jones CJ, Rikli RE. Measuring functional fitness of older adults. *J Act Aging*. 2002;(March-April):24-30.
10. Rikli RE, Jones CJ. Functional fitness normative scores for community-residing older adults, ages 60-94. *J Aging Phys Act*. 1999;7:162-181.
11. Belsky DW, Caspi A, Houts R, et al. Quantification of biological aging in young adults. *Proc Natl Acad Sci U S A*. 2015;112(30):E4104-E4110.
12. Cullinane EM, Siconolfi S, Carleton RA, Thompson PD. Modification of the Astrand-Rhyming sub-maximal bicycle test for estimating VO2max of inactive men and women. *Med Sci Sports Exerc*. 1988;20(3):317-318.
13. National Kidney Foundation. CKD-EPI CREATININE EQUATION. https://www.kidney.org/content/ckd-epi-creatinine-equation-2009. Published 2009. Accessed June 22, 2019.
14. World Health Organization. *Oral Health Surveys: Basic Methods, 2nd Ed*. World Health Organization; 1977.
15. World Health Organization. *Oral Health Surveys: Basic Methods*, *4th Ed*. World Health Organization; 1997. https://apps.who.int/iris/handle/10665/41905.
16. Glasser MF, Sotiropoulos SN, Wilson JA, et al. The minimal preprocessing pipelines for the Human Connectome Project. *Neuroimage*. 2013;80:105-124.
17. Greve DN, Fischl B. Accurate and robust brain image alignment using boundary-based registration. *Neuroimage*. 2009;48(1):63-72.
18. Robinson EC, Jbabdi S, Glasser MF, et al. MSM: a new flexible framework for Multimodal Surface Matching. *Neuroimage*. 2014;100:414-426.

© 2019 Rasmussen LJH et al. *JAMA Network Open.*

19. Glasser MF, Coalson TS, Robinson EC, et al. A multi-modal parcellation of human cerebral cortex. *Nature*. 2016;536(7615):171-178.

20. Jiang J, Liu T, Zhu W, et al. UBO Detector - A cluster-based, fully automated pipeline for extracting white matter hyperintensities. *Neuroimage*. 2018;174:539-549.

21. Wechsler D. *Wechsler Adult Intelligence Scale. 4th Ed.* San Antonio, TX: Pearson Assessment; 2008.

22. Army Individual Test Battery. *Manual and Directions for Scoring*. Washington, DC: War Department, Adjutant General's Office; 1944.

23. Sager MA, Hermann BP, La Rue A, Woodard JL. Screening for dementia in community-based memory clinics. *WMJ*. 2006;105(7):25-29.

24. Wechsler D. *Wechsler Memory Scale -- Third Edition*. San Antonio, TX: Psychological Corporation; 1997.

25. Caspi A, Houts RM, Belsky DW, et al. The p factor: One general psychopathology factor in the structure of psychiatric disorders? *Clin Psychol Sci*. 2014;2(2):119-137.

26. Touwen B, Prechtl H. *The Neurological Examination of the Child with Minor Nervous Dysfunction. Clinics in Developmental Medicine*. Vol 38. London, England: Heineman; 1970.

27. Dunn L. *The Peabody Picture Vocabulary Test*. Minneapolis, Minn: American Guidance Service; 1995.

28. Reynell J. *REYNELL DEVELOPMENTAL LANGUAGE SCALES*. London, England: National Foundation for Educational Research; 1969.

29. Bayley N. *The Bayley Scale of Infant Development*. New York, NY: Psychological Corporation; 1969.

30. Caspi A, Henry B, McGee R, T M, Silva P. Temperamental Origins of Child and Adolescent Behavior Problems: From Age Three to Age Fifteen. *Child Dev*. 1995;66(1):55-68.

31. Wechsler D. *Manual for the Wechsler Intelligence Scale for Children, Revised*. New York, NY: Psychological Corporation; 1974.

32. Elley W, Irving J. Revised socioeconomic index for New Zealand. *N Z J Educ Stud*. 1976;11:25-36.

© 2019 Rasmussen LJH et al. *JAMA Network Open.*
